# A Review of Classification Techniques of EMG Signals during Isotonic and Isometric Contractions

**DOI:** 10.3390/s16081304

**Published:** 2016-08-17

**Authors:** Nurhazimah Nazmi, Mohd Azizi Abdul Rahman, Shin-Ichiroh Yamamoto, Siti Anom Ahmad, Hairi Zamzuri, Saiful Amri Mazlan

**Affiliations:** 1 Malaysia Japan International Institute of Technology, Universiti Teknologi Malaysia, Jalan Sultan Yahya Petra, Kuala Lumpur 54100, Malaysia; nurhazimah2@live.utm.my (N.N.); hairi.kl@utm.my (H.Z.); amri.kl@utm.my (S.A.M.); 2 Department of Bio-Science and Engineering, College of Systems Engineering and Science, Shibaura Institute of Technology, Fukasaku 307, Saitama-City 337-8570, Japan; yamashin@sic.shibaura-it.ac.jp; 3 Department of Electrical and Electronic Engineering, Faculty of Engineering, Universiti Putra Malaysia, Serdang 43400, Malaysia; sanom@upm.edu.my

**Keywords:** EMG signals, isotonic contractions, isometric contractions, feature extractions, classifications, probability density functions

## Abstract

In recent years, there has been major interest in the exposure to physical therapy during rehabilitation. Several publications have demonstrated its usefulness in clinical/medical and human machine interface (HMI) applications. An automated system will guide the user to perform the training during rehabilitation independently. Advances in engineering have extended electromyography (EMG) beyond the traditional diagnostic applications to also include applications in diverse areas such as movement analysis. This paper gives an overview of the numerous methods available to recognize motion patterns of EMG signals for both isotonic and isometric contractions. Various signal analysis methods are compared by illustrating their applicability in real-time settings. This paper will be of interest to researchers who would like to select the most appropriate methodology in classifying motion patterns, especially during different types of contractions. For feature extraction, the probability density function (PDF) of EMG signals will be the main interest of this study. Following that, a brief explanation of the different methods for pre-processing, feature extraction and classifying EMG signals will be compared in terms of their performance. The crux of this paper is to review the most recent developments and research studies related to the issues mentioned above.

## 1. Introduction

The World Health Organization defines rehabilitation or rehab as the combined and coordinated use of medical, social, educational and vocational measures for training and retraining an individual to the highest level of functional ability. Physical therapy in rehabilitation assists individuals to recover as much independence as possible from neuromuscular diseases, amputation, and disability. Rehabilitation centres provide physical treatment and therapy that can help patients cope with deficits and reverse many disabling conditions that cannot be done by medical care under the supervision of therapists. Due to physical disability, assistance through an automated technical system may potentially enhance the physical activities of a patient during rehabilitation, as discovered by Mosher in the 1960s. He introduced the Human Machine Interface (HMI) as a control system and effectively demonstrated the system’s use in the mechanism of lower-limb orthoses.

Since then, advancements of HMI have extensively been developed with different types of mechanical actuators, structures and interfaces. Essentially, HMI enables humans to interact with or control the system of a machine/dynamic technical system. The term machine can also refer to a specific device, a computer program or other physical tools. In the last two decades, researchers have been developing ankle foot orthoses (AFO) to help impaired individuals walk in a more natural way. In developing AFO, special considerations were given to the algorithms with a supervisory role as those dedicated to the adaptation of different gait conditions and human motion intention recognition as discussed by Jiménez-Fabián and Verlinden [1].

**Figure 1:**
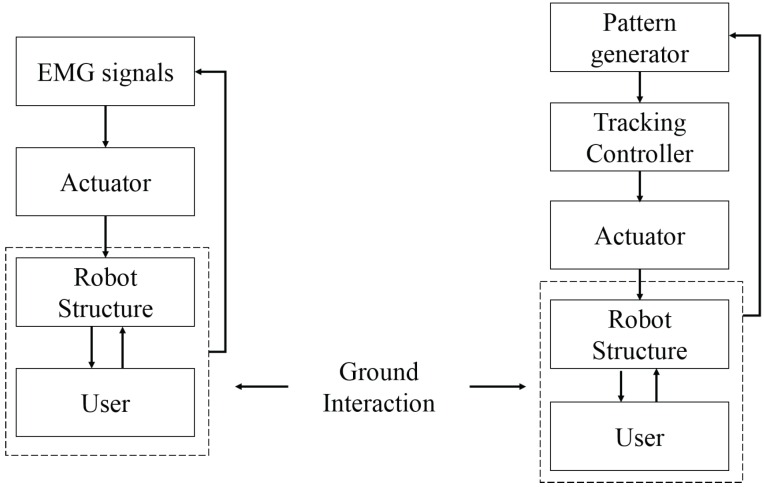
Block diagram in a control system using a gait pattern generator and EMG signals adopted from [1].

For a control system based on the periodic motion of gait, ankle behavior may be adjusted based on information about the current kinetic/kinematic state and the processes can be simplified as shown in Figure 1. Later in 2013, Dzahir et al. derived a mathematical equation of contraction model based on hip and knee joint angles to control the antagonistic mono- and bi-articular actuators of the Body Weight Support Gait Training System (AIRGAIT) [2]. Satisfactory performance was obtained when tested on a healthy subject in a robot-assisted walk test. After this study, four coordination patterns were proposed by Needham et al. to assess gait kinematics, namely, in phase with proximal dominancy, in-phase with distal dominancy, anti-phase with distal dominancy and anti-phase with proximal dominancy, which interprets the coupling angle. In clinical research, the angle movement of the pelvis, thorax, and arm kinematic are tracked by a VICON motion to deviate hemiplegic cerebral palsy patients to improve the AFO [3]. The tracking approach has also been used to determine the gait pattern of the pixel-wise binary extracted [4].

**Figure 2:**
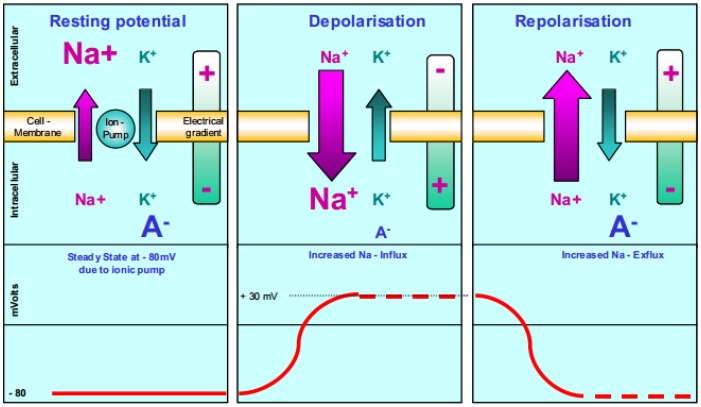
Schematic illustration of repolarization/depolarization cycle within excitable membranes.

EMG signal-based control system research is ongoing for HMI applications especially in rehabilitation [5]. Generally, EMG is an experiment-based method for evaluating and recording a series of electrical signals that emanate from body muscles. The EMG signals are formed by physiological variations in the state of muscle fiber membranes. A major factor in muscle physiology is influenced by the excitability of muscle fibers through neural control [6]. In addition, the EMG signals are based upon action potentials at the muscle fiber membrane resulting from depolarization and repolarization. Konrad states that this phenomenon can be illustrated by a model of semi-permeable membrane describing the electrical properties of the sarcolemna (the cell membrane of skeletal muscle) as shown in Figure 2.

From the figure, an ionic equilibrium between the inner and outer spaces of a muscle cell forms a resting potential at the muscle fiber membrane, which is approximately in the range of –80 to –90 mV when not contracted. Similarly, Daud et al. reports that the amplitude of surface EMG signals are in the range from microvolts, μV to millivolts, mV depending on the muscle types and conditions during the observation process [7]. This difference in potential that is maintained by physiological processes (ion pump) results in a negative intracellular charge compared to the external surface. The activation of an alpha-motor anterior horn cell (induced by the central nervous system or reflex) results in the conduction of excitation along the motor nerve. After the release of transmitter substances at the motor endplates, an endplate potential is formed at the muscle fiber innervated by this motor unit. The diffusion characteristics of the muscle fiber membrane are briefly modified and Na+ ions flow in. This causes a membrane depolarization, which is immediately restored by a backward exchange of ions within the active ion pump mechanism, also known as repolarization.

According to Reaz et al., the combination of muscle fiber action potentials from all the muscle fibers of a single motor unit is called as motor unit action potential (MUAP) [8]. This MUAP can be detected by non-invasive or invasive techniques. A non-invasive technique is applied by placing electrodes or sensors directly on the skin while an invasive approach is penetrating the needle/wire electrode into the muscle tissue to detect and record EMG signals. Notably, the non-invasive technique is preferred to measure EMG signals as this approach is free of discomfort and gives minimal risk of infection to amputees [9,10,11,12]. For surface EMG signals, the amplitude is in a range between 0 to 10 mV and the frequency range is restricted from 10 to 500 Hz.

In detecting and recording EMG signals, there are two main issues of concern that influence the fidelity of the signals;

- signal-to-noise ratio
- noise signal

The first issue examines the ratio of energy in EMG signals to energy in noise signals. In general, noise is defined as electrical signals that are not part of the desired EMG signal [8]. The second issue will be discussed extensively in the next section.

### Noises in EMG Signals

Whenever an EMG signal is being recorded from a muscle, various types of noise will contaminate it. Therefore, analyzing and classifying EMG signals is very difficult because of the complicated patterns of EMG which is influenced by the anatomical and physiological properties of muscles. The electrical noise which would affect EMG signals can be categorized into the following types:

#### Inherent Noise in Electronics Equipment

Inherent noise is electrical noise generated by all types of electronic equipment which have frequency components that range from 0 Hz to several thousand Hz [8,9]. When recording EMG signals, electrodes made of silver/silver chloride (10 mm × 1 mm) have been found to give an adequate signal-to-noise ratio (SNR) and are electrically very steady. The impedance decreases when the electrode size enlarges. Researchers are allowed to use high electrode impedances for experiments in which the statistical power is high or in which large numbers of electrodes are necessary, but are advised to switch to low electrode impedances for experiments in which statistical power would otherwise be too low. This noise can be eliminated using intelligent circuit design and high quality instruments.

#### Ambient Noise

Electromagnetic radiation is the source of this kind of noise [8]. Its amplitude is sometimes one to three times greater than the EMG signals of interest. The human body*’*s surface continuously emits electromagnetic radiation and exposure. To avoid this noise on the surface of the Earth is impracticable. Power-Line Interference (PLI) is ambient noise arising from the 60 Hz (or 50 Hz) radiation of power sources. A high pass filter can remove the interference if the frequency of this interference is high. However, if the frequency content of PLI is within the EMG signal, then it is essential to recognize the nature of the EMG signal.

#### Motion Artifact

The length of a muscle decreases when the muscle is activated. Furthermore, muscle, skin and electrodes move with respect to one another. During this time, electrodes will show some movement artifacts [8,9]. Motion artifacts cause irregularities in the data. There are two main sources of motion artifacts: (1) electrode interface and (2) electrode cable. Motion artifacts can be reduced by proper design of the electronic circuitry and set-up. The frequency range of motion noise is usually from 1 to 10 Hz and has a voltage comparable to the amplitude of the EMG. Recessed electrodes can remove the movement artifact significantly, in which a conductive gel layer is used between the skin surface and the electrode-electrolyte interface.

#### Inherent Instability of Signal

The amplitude of EMG signals is quasi-random in nature. Frequency components between 0 and 20 Hz are mostly unstable because they are affected by the firing rate of the motor units [8,9]. Because of the unstable nature of these signal components, it is considered as unwanted noise. The number of active motor units, motor firing rate and mechanical interaction between muscle fibers can change the behavior of information in EMG signals.

#### Electrocardiographic (ECG) Artifacts

Electrical activity of the heart is the foremost interfering component for EMG in the shoulder girdle, which is called an ECG artifact. This artifact often contaminates EMG signals, especially in trunk muscle electromyography [8,9]. The placement of EMG electrodes which is conducted by a selection of pathological muscle group often decides the level of ECG contamination in EMG. Due to an overlap of frequency spectra by ECG and EMG signals and their relative characteristics such as non-stationarity and varied temporal shape, it is very difficult to remove ECG artifacts from EMG signals.

#### Cross Talk

An undesired EMG signal from a muscle group that is not commonly monitored is called “crosstalk” [9]. Crosstalk contaminates the signal and can cause an incorrect interpretation of the signal information. Crosstalk depends on many physiological parameters and can be minimized by choosing the electrode size and inter-electrode distances (typically 1–2 cm or the radius of the electrode) carefully. Electrodes with a smaller surface area may reduce bipolar spacing and mathematical differentiation. Thus, the combination of these three methods decreases potential crosstalk effectively.

As various type of noises contaminate EMG signals, the process of analyzing and classifying EMG signals becomes very difficult, especially during isotonic and isometric contractions [9]. Many studies have proposed various techniques in detecting muscle activity to allow for a more standardized and precise evaluation of rehabitational, neurophysiological and assistive technological findings. Therefore, this paper will discuss techniques for features extraction and classification of surface EMG signals along with their respective advantages and disadvantages. On the other hand, probability density function (PDF) of surface EMG signals is also suggested to be applied as features in motion patterns and the potential PDF used to illustrate EMG signals will be identified. Finally, various methodologies that are used to analyze surface EMG signals will be discussed in terms of classification accuracy.

## 2. Motivation

EMG is a type of pathology, location, and etiology which can be investigated using characteristics of EMG waveforms. These techniques assist medical doctors in their diagnosis. For complicated cases, invasive methods such as muscle biopsies or more sophisticated imaging techniques such as ultrasound are preferred. Muscle contraction is the activation of tension-generating sites within the muscle fibers. A muscle fiber is excited via a motor nerve which generates an action potential that spreads along the surface membrane (sarcolemma) and the transverse tubular system into the deeper parts of the muscle fiber.

The human skeletal muscular system is primarily responsible for providing the forces required to perform various actions [13]. In the past, mechanical engineers and physicists used simulation procedures to create a model to replicate the human muscles. Hill [14] created a model that simulates human muscles which came to be known as the Hill-type model in early 1938. This model applied three mechanical elements to represent muscle behavior. They are contractile (CE), a parallel element (PE) and a series element (SE). The SE and CE are used to represent the force generated by the mechanical response and the muscle fibers to the muscle’s length changes, respectively. After this study, the Hill-type model has been widely used to estimate the muscular force generated by humans. In 2001, Rosen and his colleagues presented a method to control a powered exoskeleton arm system using the model [15]. The motion of flexion and extension muscles of the elbow joint was estimated based on the Hill-type model and was used to control the robot arm system. To apply the Hill based model, Fleischer et al. [16] proposed a direct force control (DFC) and dynamic human body model (DHBM) to achieve exoskeleton robot control in both the lower extremities and hands.

Unfortunately, the Hill-type model is a complex one as many parameters such as muscle fiber length or muscle contraction velocity. This internal information varies according to each subject and a calibration procedure is necessary to obtain the information in a way to control the prostheses or robots. In addition to building a muscle model, machine learning algorithms can be employed to recognize a user’s intention based on the motion patterns of EMG signals. As a result, the user can possibly control the assistant device upon recognition of the results intuitively. In 2014, Naeem had compared his proposed method based on the Fuzzy Logic theorem with the Hill-type model [17]. The general model extracted muscle force features from EMG signals and the model is almost similar with the Hill-type model.

**Figure 3:**
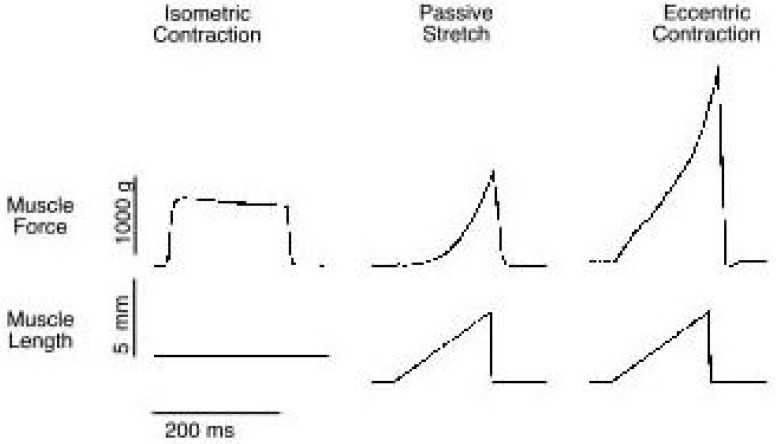
Different types of muscle contractions based on muscle force and length.

In classical neurological EMG, an artificial muscle response due to external electrical stimulation is analyzed in static conditions. Unlike the classical neurological EMG, the focus of kinesiological EMG can be described as the study of neuromuscular activation of muscles within postural tasks, work conditions, functional movements and training/treatment regimes. The fundamentals behind most of the common resistance-training exercises can be categorized into two classes, namely isotonic and isometric contractions as shown in Figure 3. These two types of resistance contractions are performed very differently and have different purposes. Analysis during isotonic contractions is most common for strength and athletic goals, meanwhile isometric contractions are most often used for physical rehabilitation. The details of both contractions will be discussed in the following sections.

### 2.1. Isotonic

Isotonic contractions involve muscular contractions against resistance in which the length of the muscle changes. In other words, this type of contraction generates force by changing the length of the muscle and it can be either concentric contractions or eccentric contractions. By pairing both contractions, the movements will create a dynamic contraction produced during dynamic exercise. Dynamic exercise is any exercise that involves joint movement, such as a dumbbell bicep curl exercise.

#### 2.1.1. Concentric Contractions

Contractions that permit the muscle to shorten are referred to as concentric contractions [18]. More specifically, concentric contractions occur when the tension stays the same while the length is getting shorter and the energy is fluctuating. In particular, the muscle begins to shorten when a muscle is activated and required to lift a load, which is less than the maximum tetanic tension it can generate [19]. This can be seen through the raising of a weight during a bicep curl. By performing a series of constant velocity shortening contractions, a force-velocity relationship can be determined.

#### 2.1.2. Eccentric Contractions

Contrary to concentric contractions, the length of eccentric contractions are longer, affecting the muscles to elongate in response to a greater opposing force. As the load on the muscle increases, it finally reaches a point where the external force on the muscle is greater than the force that the muscle can generate. Thus, even though the muscle may be fully activated, it is forced to lengthen due to the high external load. There are two main features to note regarding eccentric contractions:

- Absolute tensions achieved are very high relative to the muscle’s maximum tetanic tension generating capacity.
- Absolute tension is relatively independent of lengthening velocity.

This suggests that skeletal muscles are very resistant to lengthening. The basic mechanics of eccentric contractions is still a source of debate since the cross-bridge theory that conveniently describes concentric contractions is not as successful in describing eccentric contractions.

Eccentric contractions are currently a very popular area of study for three main reasons: First, much of a muscle’s normal activity occurs while it is actively lengthening, so eccentric contractions are physiologically common, for instance by walking [20,21]. Second, muscle injury and soreness are selectively associated with eccentric contraction. Finally, muscle strengthening may be best using exercises that involve eccentric contractions. Therefore, there are some very fundamental structure-function questions that can be addressed using the eccentric contraction model. Eccentric contractions have very important applications that can therapeutically be used to strengthen muscles.

### 2.2. Isometric

**Figure 4:**
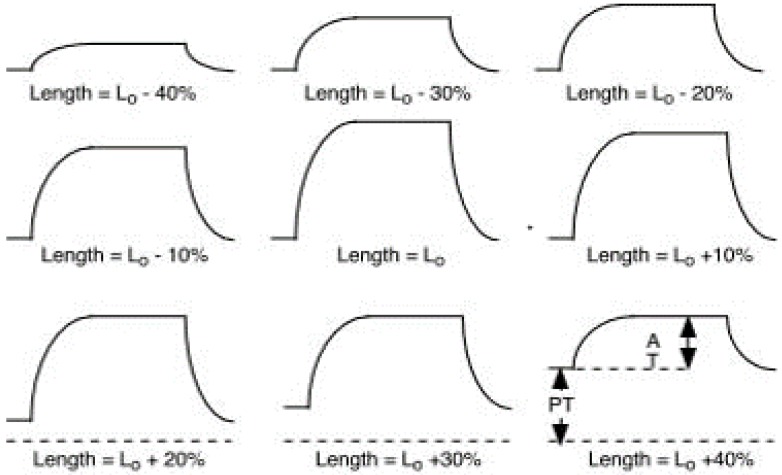
A series of isometric contractions performed at different muscle lengths.

Contrary to isotonic contractions, isometric contractions create no change in muscle length but tension and energy are fluctuating. This action causes muscles to produce force. The force generated during an isometric contraction is wholly dependent on the length of the muscle while contracting as can been seen in Figure 4. In other words, isometric contractions are done in static positions where the joint angle and muscle length do not change during contraction. An isometric contraction is typically performed against an immovable object. This can be seen through side planks or wall-sits.

The EMG signals recorded in isotonic conditions are widely used in clinical applications to classify neuromuscular diseases [22] and muscles fatigue [23]. Meanwhile, a large and growing body of literature analyzes EMG signals in isometric and isotonic contractions to control assistive robots, lower-limb orthoses and exoskeletons as reviewed in [24,25]. Several studies conducted had segmented EMG signals obtained during isotonic contractions using adjacent or overlapped windowing techniques for further analysis [26,27,28]. Nevertheless, Tsai et al. reported that EMG signals collected from the same type of muscle contraction is preferable, whether isotonic or isometric based on its experimental results for the purpose of controlling the exoskeleton robot [29]. Therefore, the comparable results in features extraction corresponding to isotonic and isometric contractions of EMG signals via skin surface is investigated in this study. In addition, to use EMG signals in controlling HMI, advanced mathematical and statistical methods such as probability density function (PDF) is necessary to describe the amplitude of EMG signals as discussed in [30,31]. Thus, an overview of techniques in EMG signal analysis and potential PDF proposed in literature during isotonic and isometrics conditions will inspire researchers in both clinical and engineering areas.

## 3. Research Methodology

**Figure 5:**
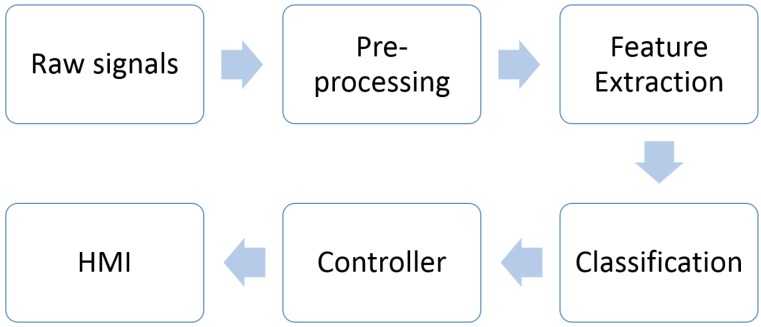
An overview in developing EMG control systems.

Generally, the process of developing assistive devices based on EMG signals is presented in Figure 5. Three main cascaded modules should be carefully considered, consisting of pre-processing, feature extraction and classification. Numerous approaches have been proposed to achieve better performance of classification. In order to obtain higher classification accuracy, the selected features are the main kernel used in analyzing EMG signals [28]. The main purpose of this paper is to examine the accuracy of the classification system conducted by previous researchers. Firstly, the methods applied in pre-processing, feature extraction and classification for automated EMG analysis will be reviewed. Then, the EMG dataset used in the literature will be identified. A comparison of techniques and muscle contractions will be discussed, followed by future trends. Lastly, we summarize the methods to analysis EMG signals and conclude the outcome of previous research.

## 4. Automated EMG Analysis

### 4.1. EMG Signal Preprocessing

Various techniques for handling data of EMG signals before the feature extraction and pre-processing stages (e.g., data segmentation, filtering and rectification) will be used to improve the accuracy and response time of the data controller. Initially, data will be segmented from the raw EMG signals. For each divided segment which has been filtered and rectified, a feature set (Section 5) will be computed and then be fed to the classifier (Section 6), and these processes are continuous. For data segmentation, the windowing technique and data length are two main points that need to be considered.

Englehart and Hudgins pointed out that the different lengths of EMG data effect its classification error [32]. This statement was proven by Farina and Merletti as the performance of classifier is degraded using a segment length that is less by 128 ms, leading to high bias and variance of features [33]. Similar with a study conducted in 2013, the accuracy of classification increases when the segment length increases from 125 to 500 ms [27,34]. This is because a larger segment provides additional information and yields small bias and variance in the estimation of the feature. This segment condition provides high accuracy and can be operated in real-time applications for the upper limb [35]. In [28], the sample length of EMG data is set to 256 ms during the beginning of the movement as this span contains the information of movement data. For prosthetic limb control, the response time should be less than 300 ms in order to reach the real-time constraints [36].

There are two main methods used for data windowing, namely adjacent and overlapping. Adjacent windowing is where adjacent disjointed segments with predefined length uses feature extraction and classification after a certain processing delay, τ. The τ is the time required to calculate the feature and classify the data. The drawback of this technique is that the τ will cause the processor to stay in an idle condition during the remaining time of the segment length [35]. This matter is overcome with the overlapped windowing technique, where the new segment slides over the current segment and the increment time is less than the segment length. However, the performance of the overlapped segmentation has no improvement in classification accuracy, but it is significant to be employed for large segments (greater than 200 ms) in order to avoid delays in time [27].

To overcome various noises mentioned in the previous section, EMG signals need to be filtered to reduce the artifacts. Balbinot and Favieiro used a band pass filter of 500 Hz cutoff frequency for high pass filter and a low pass filter of 20 Hz cutoff frequency to reduce motion artifacts [13]. For the elimination of ECG artifact from surface EMG signals, Yeom and Yoon [37] compared the performance of an adaptive filter which is a reliable and efficient tool for mixed and varied patterns of transient, short and long lasting dystonic contractions as proven by Luca et al. [38], bandpass filtering methods, and mathematical morphology operator (MMO) methods. Even though the adaptive filter produces higher sensitivity which is associated with leaving EMG signals in ECG signals, adaptive subtraction method is somewhat effective to remove ECG artifact from contaminated electromyogram signals and has an acceptable result [39]. In detecting muscle activation patterns for the upper limb, raw EMG signals were filtered using an adaptive whitening filter [40] and high-pass finite impulse response filtered with 100 taps at a cutoff frequency of 20 Hz [11].

For lower limbs, most studies filtered EMG signals using a Butterworth filter with different orders and cutoff frequencies. Nadzri et al. [10] applied a low-pass filter with a cutoff frequency 6 Hz, Kendell et al. [41] used a 6th order low pass with a cutoff frequency of 5 Hz and Al-Angari et al. [42] used a band pass with a cutoff frequency from 5 Hz to 500 Hz. Other studies reduced the noise in EMG signals using a high-pass filter with a 500 Hz cutoff frequency to reduce motion artifacts and a low pass filter of 20 Hz cut-off frequency [13]. Nevertheless, it remains very difficult for the noise to be removed clearly [9].

Thus, there is little attention in the literature for the filtering stage. The selection methods for the pre-processing stage are determined according to its applications. Yet, most of the studies highlighted the classification accuracy in the analysis of EMG signals for motion patterns. In addition to successfully recognizing the motion patterns, a proper machine learning method is critical. Current classification algorithms mostly tested with EMG signals on transient or stationary scenarios separately were reported. The relationship between both states is thoroughly examined. The next section will discuss the types of muscle contractions for EMG signals.

### 4.2. EMG Feature Extraction and Selection

In signal processing analysis, feature extraction plays a critical role to achieve a better performance of classification for motion pattern recognition. This process involves the transformation of raw EMG signals into a feature vector. Generally, features in the analysis of EMG signals can be divided into three categories, including time domain (TD) features, frequency domain (FD) features and time-frequency domain (TFD) features [29,43,44]. For TD features, the features are evaluated based on signal amplitude that varies with time. The amplitude of the signal depends on muscle conditions and types during the observation process. To keep the computational complexity low, most previous studies had focused on TD features. In addition, these features do not require additional signal transformation. Unlike TD features, FD features contain the power spectrum density (PSD) of the signals and are computed by parametric methods or a periodogram. On the other hand, a combination information of time and frequency are defined as TFD features. TFD features can characterize varying frequency information at different time locations, providing plentiful non-stationary information of the analyzed signals. Oskei and Hu had illustrated the key parameters in each domain of signal analysis [45].

In 1993, five TD features were proposed by Hudgins et al. [46]: mean absolute value (MAV), mean absolute value slope, slope sign changes (SSC), zero crossing (ZC) and waveform length (WL). According to Tsai et al., the time taken to extract the features set is approximately 10 ms for 200 ms of sampled data collected for normal and amputee subjects during dynamic and static contractions of the arm [29]. The ZC and SSC features in TD represent rough FD information but do not involve converting EMG signals to FD. In the detection of hand motions, Ahsan et al. extracted EMG signals using MAV, ZC, SSC, root mean square (RMS), variance (VAR) and standard deviation (SD) [47]. Extended work was conducted in 2013 by adding one more feature, WL, and the feature is fed as an input to the classifier. In the same year, another TD feature, namely maximum amplitude (MAX) is used along with SD and RMS to interpret EMG signals within hand-lifting three different loads. SD had the best overall performance compared to MAX and RMS [7]. Furthermore, RMS and MAX features are the better ones that can be used with SD for a useful feature vector.

In 2014 [48], the complexity of EMG signals of patients after stroke during 20 sessions of robot-aided rehabilitation training was investigated using two indexes: Fuzzy approximate entropy (fApEn) features and maximum voluntary contraction (MVC). Other TD features such as skewness (Skew) [45], Kurtosis (Kurt) and moving approximate entropy (moving ApEn) were initially employed by Ahmad and Chappel in 2009 for prosthetic hand applications. Moving ApEn effectively recognizes the stages of contraction (e.g., start, middle, end) based on surface EMG signals of flexor carpi ulnaris and extensor carpi radials muscles [35]. The research clarified that using moving ApEn to extract features in clinical processes is promising.

Work done by Balbinot and Favieiro showed that features in TD, specifically RMS is possible to obtain for each of the eight channels and these values can be used as inputs to the classifier for the windowing signal to occur at the instant when a movement occurs [13]. Similarly, RMS appears to be the best parameter compared to MAV, MAX, SSC, ZC and WL as it provides a quantitative measure for electrode selection [41], thus delivering the best performance for facial gestures of EMG signals [49]. On the other hand, Integrated EMG (IEMG) features used to determine an increase in signal period, power and amplitude reflects a higher muscle fiber recruitment for a fixed external force [19]. Several studies have explored potential TD features that can be calculated based on raw EMG time series as shown in Table 1.

Only a couple of studies had used FD as features in the motion pattern recognition. Spectral or frequency domain features are mostly used in the assessment of muscle fatigue and motor unit recruitment analysis as discussed by Al-Mulla et al. [19]. Changes in EMG signals in the FD relate to the median power frequency (MPF), which varies due to a shift towards lower frequencies such as a relative decrease in high-frequency signal power, a small increase in low-frequency signal power, an increase in high-frequency spectrum slope or a decrease in low-frequency spectrum slope. In clinical practice, power spectral analysis such as mean power frequency (MPF) of EMG signals provides information regarding the complex changes in muscular and neural signals induced by stroke survivors [54]. The study showed that a majority of stroke subjects have lower MPF in their paretic muscles than in their contralateral muscles at matched isometric contraction force.

On the other hand, PSD, mean frequency (MNF) and median power frequency (MNP) of the power spectrum are usually applied as indices to characterize EMG signals, especially for muscle contractions [55]. The conventional features had been modified by Phinyomark et al. in a way that robust features can be extracted to track the progression of fatigue over time [56]. They modified the mean and median frequency by calculating the mean and median of amplitude spectrum instead of power spectrum, which are defined as modified mean frequency (MMNF) and modified median frequency (MMDF). The MNF, median frequency (MDF), bandwidth (BW) and Normalized spectral moments (NSM) are extracted to detect muscle fatigue for upper limbs [23]. Farina and Merletti defines the PSD of a wide sense stationary stochastic process as a Fourier transform of the autocorrelation function of the EMG signal [33]. All the possible FD features to be extracted are shown in Table 2.

Several studies have investigated the performance of TD and FD features. Phinyomark et al. had carried out a comparison between the performance of twenty-seven TD features and eleven FD features to discriminate hand movements. EMG signals were obtained at a constant force and static contraction. As a result, TD features were superfluid and redundant based on the scatter plot of features, statistical analysis and classifier [28]. Even though the time consumption and dimension for TD features were faster and smaller than other features, recognition performance was not satisfactory as claimed by Tsai et al. [29].

Previous studies have indicated that neural control strategies of isometric and isotonic contractions differ. For instance, during isotonic contractions, numerous motor units (MUs) showed lower recruitment thresholds [58,59], which implies that forces generated by muscles during various types of contractions must be considered differently. In motion recognition applications, normalization is a crucial step and changes in EMG amplitude can influence the normalization result, affecting recognition performance. Conversely, the behavior of MUs during dynamic contraction also differs from their behavior during isometric contraction. Thus, the spectrum of EMG signals and muscle activation pattern of these two types of contraction might be dissimilar [29]. In TD, the magnitude of EMG signals during isotonic contraction can differ from and be greater than those during isometric contraction [26].

The work of Kendell et al. [41] represents a study of electrode-pair selection based on the characteristics of EMG signals using six TD features and five FD features. In the study, TD provides a more consistent method than FD features for electrode selection. Similar to a study conducted in 2013 by Phinyomark et al., TD features yield a better performance than FD features for long term. Nonetheless, TD features assume data as a stationary signal, which is inappropriate to be employed for EMG signals as these signals are non-stationary [23]. Ramirez and Hu mentioned that these features give a measure of frequency, waveform amplitude, and duration within some limited parameters [24].

A multiple feature performance had been investigated. In 2006, Oskoei et al. combined both TD and FD features into a feature vector. The result implies that using TD features alone cannot provide satisfactory accuracy for recognizing the four motion patterns [60]. Statistical analysis shows that the four motion patterns represented by AR, RMS, and STFT-ranking feature (β=5) exhibit statistically significant differences during both isotonic and isometric contractions, implying that applying multiple features can obtain more satisfactory recognition performance [29]. However, features based on MNF, MDF, PKF, MNP, TTP, Spectral Moments, FR, PSR and VCF are not good in EMG signal classification [28].

In the quest to improve classification accuracy, an ensemble of TFD features are proposed in [44] to overcome the limitation of TD features, which is applicable for stationary signals [35]. Investigation on the performance of TFD (e.g., Wavelet and Wavelet Packet Transform) and TD features for the upper limb was conducted by Englehart et al. Based on classification error, a wavelet packet transforms features, thus yielding a lower percentage of classification error at 6.25% compared to TD features at 9.25% [44]. While the tiling of the STFT and the WT is fixed, the tiling of the WPT may be adapted to suit a particular application. TFD features have localized the energy in time and frequency, allowing for an accurate description of the physical phenomenon. This statement is supported by Basu et al. as they demonstrate the ability of TFD features like wavelet transform and STFT to track time-varying frequencies and mode shapes [61].

In 2016, Guo and Karem proposed a new output-only non-stationary system identification (SI) framework based on instantaneous or marginal spectra derived from TFD features to identify time-varying system properties [62]. Surprisingly, the major problem of TFD features is high dimensionality and high-resolution of feature vectors [9]. To encounter the complexity of TFD features, dimensionality reduction is implemented to reduce the dimensionality of the data while maintaining its discrimination capability [24,35]. According to Englehart [63], there are two main strategies for dimensionality reduction:

- Feature projection
- Feature selection

Feature projection methods attempt to determine the best combination of the original features to form a new feature set which is generally smaller than the original one [45]. The other strategy chooses the best subset of the original feature vector according to specific criteria for judging whether one subset is better than another [24]. Moreover, the selection of a feature vector ought to be carefully considered. Although [28] explores the quantitative comparisons of feature vectors for numerous specific EMG signal classifications, from a redundancy point of view, TFD features need more computing time to extract the features. Table 3 summarizes an example of TFD features based on literature.

### 4.3. Probability Density Function

Special attention is paid to probability density function of EMG signals in this paper. Statistical modeling is among the most important factors for many engineering problems as stated by Carrillo et al. [64]. Mathematical modeling of interesting signals is the ordinary approach to improving our understanding of intrinsic biological phenomenon [65]. Furthermore, the mathematical parameters of surface EMG signals are influenced by many factors such as level of effort, fatigue, muscle length, muscle fiber architecture and electrode positioning. Due to these aspects, Rassol et al. have characterized surface EMG signals of lower extremity muscles by means of statistical properties [66], which are relevant in signal modeling.

Investigation concerning the best PDF to be used to describe the distribution of sampled surface EMG signals becomes relevant because it can help improve algorithms of onset detection applied in neuroprosthesis [67], biofeedback [68], image processing [69,70,71] and extreme climates [72,73,74]. For a given dataset, the estimation of underlying PDF for pattern recognition and machine learning has been used for many years by statisticians and engineers. They used density estimators as a tool to draw inferences from physical data in social and computer sciences [75]. This approach is not only from the desire to accurately characterize stochastic events like surface EMG signals, but also from the fact that distributions are the central models utilized to derive sample processing theories and methods.

A probability distribution is defined as a table or an equation that links each outcome of a statistical experiment with its probability of occurrence. The probability of a random variable falling within a particular range of random values is given by the integral of the variable*’*s density over that range. The PDF is a function that describes the relative likelihood of the random variable as a given value. In other words, the PDF is nonnegative and its integral over the entire space is equal to one. Most studies conducted have focused on isometric contractions. To our knowledge, there is no consensus in the literature about the appropriate PDF to describe the behavior of the characteristics of EMG signals.

The EMG signals are stochastic [23,76,77] as the signals are non-deterministic and there are distinct patterns at three states of contraction [12]. In other words, it can be modeled as a random process where its density/amplitude is typically assumed to be Gaussian/Normal based on the relationship between muscle contraction and myoelectric activity as defined by Hogan and Mann in the 1980s [43]. This set of assumptions represents a combination of frequency content of subcutaneous myoelectric activity and the filtering effect of transmission through a non-invasive approach subsequently multiplied by a static, nonlinear function of muscle force. A physical situation corresponding to this model is a non-fatiguing condition where the muscle contracts isometrically.

In 1987, Hunter et al. [78] examined the sampled distribution of surface EMG signals collected from the biceps brachii muscle during isometric and non-fatiguing contractions at 30% of the maximum voluntary contraction (MVC). After performing a graphical comparison, the authors reported that the shape of the experimental sample distribution was considerably different from a Gaussian one, being more peaked than a normal PDF around zero means. A similar result was obtained by Bilodeau et al. [79], even though the percentage of MVC were varied at 20%, 40% 60% and 80% along with non-fatiguing and isometric contractions. The signal presents a non-Gaussian sample distribution in general which is more peaked around the zero mean based on the Shapiro-Wilk test. In addition, they observed that the peaking of sample distribution was less pronounced at higher levels of muscle contraction.

In the same context of detecting EMG signals, an assumption by Hogan and Mann had been re-examined theoretically and experimentally by Clancy and Hogan in 1999 [77]. In this study, experimental data from constant-force, constant-angle, non-fatiguing contractions falls between the Gaussian and Laplace distributions. However, on average, Gaussian density showed a better fit with surface EMG signals based on the differences between histograms obtained from the experimental data and theoretical PDF. Based on the figure, dotted line indicates the Laplacian density and dashed line indicates the Gaussian density. Meanwhile, the experimental density (solid line) represent the average of 660 recordings and the shaded region indicates one standard deviation for above and below the average.

In particular, Carrillo et al. discussed that the Gaussian distribution is too light-tailed to model signals and contains noise that exhibits impulsive and non-symmetric characteristics [64]. Therefore, they developed a generalized Cauchy distribution theory-based approach that was guaranteed to solve challenging problems formulated in a robust fashion. Similarly in 2015, the observation noise of EEG, EOG, and EMG signals were modeled using Cauchy distribution [80] as the noise has a heavy-tailed distribution.

Due to controversy in the literature, Rose et al. investigated the suitable PDF in modeling sample distribution of surface EMG signals during isometric contractions. They argued that such metrics used in [77] is not supported in probability theory because it is supposed that the area below any PDF should be unitary [65]. Moreover, the shape of surface EMG signals exhibit more peaks than a PDF of Gaussian around zero means. This finding suggests that PDF of Logistic is preferred as the distribution as it produces the minimum mean absolute error (MAE) between the histogram obtained from the experimental data and the PDF compared to Normal, Laplace, Logistic and Cauchy distributions as shown in Table 4. Within the same year, work done by Thongpanja et al. indicated that the PDF of surface EMG signals tend to be Gaussian at high-level of muscle contraction and tend to be Laplacian at the low-level contraction of biceps brachii [76].

In 2015, a research conducted by Nazmi et al. [81] revealed potential to implement Extreme Value Theory (EVT) as biosignals exist at abnormally low or high values of data, categorized as extreme events according to Markose and Alernton [82]. A Generalized Extreme Value (GEV) distribution is found to be the most appropriate distribution for describing the raw data of EMG and EEG signals. This is based on a minimum error produced by two Goodness-of-Fit (GOF) tests, namely, Kolmogorov-Smirnov statistic and Anderson-Darling statistic compared to the Generalized Pareto (GP) and Exponential (EXP) distributions. Nevertheless, this approach is applicable to determine the maximum value only which is mostly conducted in image processing [69,70,71] and climate extremes [72,73,74]. For invasive methods, the structurally developed model developed by De Luca [83] is more adequate to describe EMG signals.

Some authors have investigated the suitability of Normal distribution in describing the sample distribution of intramuscular EMG signals and concluded that it might be fitted by some other peaked PDF. In the mid-1970s, Milner-Brown and Stein [84] found that the sample distribution of intramuscular EMG signals recorded on first dorsal interosseous muscle in a condition of constant force (isometric contraction) and angled contraction presented a pattern that is sharper and peaked around the zero mean than the one predicted by PDF of Gaussian. Indeed, this peak in the sampled distribution seemed to be less pronounced at higher muscle force levels. In contrary, Parker et al. [85] observed that intramuscular EMG signals collected from the biceps brachii muscle during two different low levels of muscle contraction are reasonable to be modeled using a Gaussian distribution based on a comparison performed graphically. Table 5 summarizes the possible PDF of EMG signals during isometric contractions.

**Figure 6:**
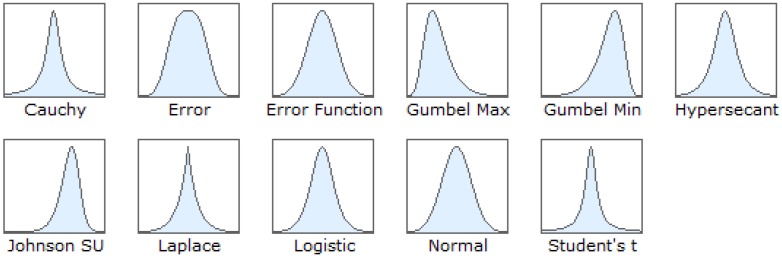
Basic shapes of each distribution.

Basically, the PDF of Cauchy, Gaussian, Laplace and Logistic are categorized as unbounded distributions [86] and continuous probability distribution. Figure 6 shows the basic shape of different types of distribution. The PDF of distributions consist of two or three parameters, namely location, scale and shape, which depends on the type of distribution. The details of each PDF will be explained.

The Cauchy distribution is often used in statistics as the canonical example of a *’*pathological*’* distribution since both its mean and its variance are undefined. The Cauchy distribution has the probability density function given by [86]:

(1) $$f\left(y\right)=\frac{1}{\pi \alpha (1+{\left(\frac{y-\varepsilon }{\alpha }\right)}^{2})}$$

where ε is a location parameter, specifying the location of the peak of the distribution and α is the scale parameter which represent the half-width at half-maximum with parameter space −∞<ε<∞,α>0,−∞<k<∞. The amplitude (or height) of the Cauchy function us given by

(2) $$Amplitude=\frac{1}{\pi \alpha }$$

The Gaussian (or Normal) distribution is remarkably useful because of the central limit theorem. The normal distribution is sometimes informally called the bell curve. However, many other distributions are bell-shaped (such as Cauchy’s, Student’s, and logistic). The terms Gaussian function and Gaussian bell curve are also ambiguous because they sometimes refer to multiples of the normal distribution that cannot be directly interpreted in terms of probabilities. The probability density of the normal distribution is [86]:

(3) $$f\left(y\right)=\frac{\text{exp}(-\frac{1}{2}{\left(\frac{y-\varepsilon }{\alpha }\right)}^{2})}{\alpha \sqrt{2\pi }}$$

where ε and α are the location and scale parameter respectively.

The Laplace distribution is also sometimes called the double exponential distribution because it can be thought of as two exponential distributions (with an additional location parameter) spliced together back-to-back, although the term *’*double exponential distribution*’* is also sometimes used to refer to the Gumbel distribution. The difference between the two independent identically distributed exponential random variables is governed by a Laplace distribution, as in a Brownian motion evaluated at an exponentially distributed random time. Increments of a Laplace motion or a variance gamma process evaluated over a time scale also has a Laplace distribution. The probability density of the Laplace distribution is [86]:

(4) $$f\left(y\right)=\frac{\lambda }{2}\text{exp}\left(-\lambda |y-\varepsilon |\right)$$

where λ and ε are continuous inverse scale and location parameter respectively with parameter space λ > 0 and −∞<y<∞.

The cumulative distribution function of logistic function appears in logistic regression and feedforward neural networks. It resembles the normal distribution in terms of shape but has heavier tails (higher kurtosis). The PDF of the logistic distribution is given by [86]:

(5) $$f\left(y\right)=\frac{\text{exp}-(\frac{y-\varepsilon }{\alpha })}{\alpha (1+\text{exp}\left(\frac{y-\varepsilon }{\alpha }\right))}$$

where ε and α are the location and scale parameter respectively.

The GEV distribution is a family of continuous probability distributions. This distribution was introduced by Jenkinson in 1955 [87]. GEV is developed within the EVT to combine the Gumbel, Fréchet and Weibull families. Let $y_{1},\ldots ,y_{n}$ denoted the independent sampled distribution EMG signals, the probability density function of GEV is [74];

(6) $$f\left(k,\varepsilon ,\alpha \right)\left(y\right)=\frac{1}{\alpha }{\left[1+k\left(\frac{y-\varepsilon }{\alpha }\right)\right]}^{-1\frac{-1}{k}}\text{exp}-[1+k\left(\frac{1}{\alpha }{\left[1+k\left(\frac{y-\varepsilon }{\alpha }\right)\right]}^{\frac{-1}{k}}\right)]$$

where ε is the location, α is the scale and *k* is the shape parameters with parameter space −∞<ε<∞,α>0,−∞<k<∞ respectively.

The PDF of distribution consists of two or three parameters, namely, location, scale and shape, which depend on the types of distribution. The location parameter represent the peak of the sampled data of EMG signals and scale parameter illustrate the position of the peak in the domains. A model of univariate data set with a probability distribution can be developed by estimating the parameters of the distributions. The method of Moments (MOM) and Maximum Likelihood Estimation (MLE) are examples of approach methods for parameter estimation. In hydrology applications, parameter estimations via MLE method is the best method due to its all-around utility and adaptability to model change for rainfall [88], while is incapable to obtain the parameter estimation for a small sample in a flood frequency analysis [89]. MLE was proved to be very effective in de-noising magnetic resonance images [71] and efficiently transform quantized observations into multiple noise environments [90]. Morever, Xu and Lee suggested that MLE is consistent and asymptotically and normally distributed for a Tobit model [91]. Despite that, in [77], the MAV processor is the MLE of EMG amplitude for the Laplace model.

In a probability theory, the term parameter estimation refers to the process of using sample data to estimate the parameters of distribution. To estimate parameters using the MLE technique, a random variable, *y* and the PDF conditioned on a set of parameters, θ, denoted as f(y|θ) are used. The joint density of *n* independent and identically distributed observations from this process is the product of individual densities given by [74]:

(7) $$\begin{array}{ll} f\left(y_{1},y_{2},\ldots y_{k}|\theta \right) & =\Pi_{i=1}^{n}f\left(y_{1}|\theta \right)\\ & =L(\theta |y) \end{array}$$

This joint density is the likelihood function, defined as a function of the unknown parameter vector, θ, where Y is used to indicate the collection of sample data. It is usually simpler to work with the log of the likehood function:

(8) $$lnL\left(\theta |y\right)=\sum_{i=1}^{n}lnf\left(y_{i}|\theta \right)$$

The value of parameters will be obtained by differentiating the (9) with respect to each parameters of distribution and equating the resulting to zero. However, this step does not stop here, since the iterative procedure needed to solve the numerically. Newton’s method will be used in this procedure as in equation below;

(9) $$a_{n+1}=a_{n}-\frac{F(a_{n})}{F^{\prime }\left(a_{n}\right)}$$

As a result, the estimated value of parameters will be used to represent the shape distribution of the EMG signals.

### 4.4. EMG Classification

The information extracted from the EMG signals will be then fed into classifier to map different patterns and match them appropriately. Classifiers should be deployed to distinguish different categories of the features extracted. Then, the obtained categories are going to be applied in the next stage as control commands for the controller. Several techniques are deployed to classify EMG data such as artificial neural networks (ANN), Bayesian classifier (BC), fuzzy logic (FL), multilayer perceptron (MLP), support vector machines (SVM), linear discriminant analysis (LDA), hidden Markov models (HMM) and K-nearest neighbor (KNN). Recently, many researchers have shown interest in effective means of classifying the pattern of EMG signals.

In 1999, Englehart et al. [44] stated that the performance of feature extraction and dimensionality reduction is dependent upon the capabilities of a classifier. Statistical classifiers, also known as LDA and MLP had been used in the study to classify hand motions. The best performance is exhibited using LDA with a classification accuracy 93.75% when using a PCA reduced feature set. They also discovered that the MLP enjoys an advantage over the LDA for being capable of prescribing nonlinear class boundaries so as to encompass the capabilities of the LDA. Two years later, a LDA classifier performs well than a MLP classifier for the TFD based features sets [36] as mentioned in [44]. The LDA does not require heuristic specifications of its architecture or training algorithm, yet it consistently performs very well. This, presumably, is due to the fact that the PCA dimensionality reduction has an effect of linearizing the discrimination task of the classifier. In 2013, Phinyomark et al. compared the performance of LDA, random forests (RFS), decision tree (DT), k-nearest neighbor (KNN), support vector machine (SVM), and multi-layer perceptron neural networks (MLP-NN) quadratic discriminant analysis (QDA) [27] to classify ten upper limb motions. As a result, LDA gained 98.87% of classification accuracy based on TD features. However, works done by Khushaba and Al-Jumaily produced about 99% classification accuracy by using MLP to classify human forearm motions based on TFD features [92].

On the other hand, an ANN approach is suitable for modeling nonlinear data due to its ability to cover the distinctions among different conditions like hand motions (left, right, up and down). The overall performance for a single trial has been found at 89.2% with an average success rate of 88.4% based on TD features as conducted by Ahsan et al. [47]. Yet, the precision of ANN outputs is always limited to the least square errors as discussed in [93]. Xie et al. claimed that the training time of ANN is quite long and the training data have to be chosen over an entire range where variables are expected to change. In addition, it is difficult to determine the proper size and structure of an ANN to solve a given problem.

**Figure 7:**
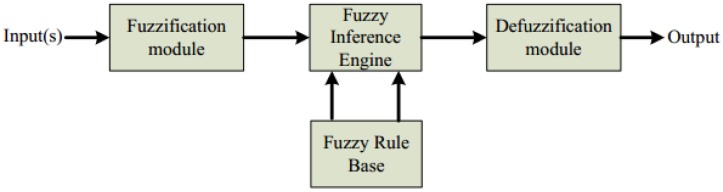
General block diagram for FL systems [35].

Another technique that has been applied in the classification of EMG signals is the FL system. FL provides a simple way to achieve a definite conclusion just upon using imprecise input information which mimics a user’s intent to make a decision according to biosignal characteristics which is not always repeatable. FL has the advantage for control techniques in biosignal processing [35]. Essentially, the FL system consists of three stages which are input, processing and output, as shown in Figure 7. In the input stage, also called as the fuzzification module, the signal features used will be converted into a state (for instance, up and down hand motions) that is called membership function (MF) and truth values. In the processing stage, also known as the inference rule base stage base, all information will be processed based on the rules generated in IF-THEN form. An appropriate rule will be invoked at this stage, generating the result for each rule, which then combines the result of the rules. The output stage, which also has its own membership function will then convert the combined results obtained at the previous stage into a final output value. This procedure is called defuzzification. Even though the correct set of fuzzy rules and membership functions are difficult to be determined to describe system behaviour in FL algorithms, a study conducted by Ahmad and Chappel with the aim to detect the stages of contraction on wrist muscle gained a classification accuracy of 97% when using FL as a classifier [35]. In 2015, Xie et al. [93] mentioned that although studies within the FL systems utilizes the IF-THEN rules which are capable to emulate human decision making more closely than the other classifiers, there are critiques concerning FL algorithms. They clarify that FL approaches require more system memory and processing time as the use of FL limits system knowledge more in the rule base than in the membership function base of fixed geometric-shaped membership functions.

Despite that, neuro-fuzzy systems computing enables us to build a more powerful intelligent decision-making system by combining the advantages of an artificial neural network with the fuzzy modeling of imprecise and qualitative knowledge [94]. Hussein and Malcolm [95] applied a neuro-fuzzy classifier to classify the intention of a paraplegic person to stand up or sit down according to single-site EMG signals obtained from the triceps and biceps brachii muscles for electrical stimulation orthosis purposes. This neuro-fuzzy hybridisation was functionally based on the Surgeno-type fuzzy rule base along with a radial basis function (RBF) neural network under some constraints to allow for the system to learn from the training data. As a result, this classifier is capable of identifying 28 sitting and 29 standing EMG signals out of 60 EMG signals by using seven bell-shape membership functions and 30 rules. ANFIS is a type of neural network structure based on the Takagi–Sugeno fuzzy inference system. It has the potential to capture the benefits of both techniques in a single framework by integrating both neural networks and fuzzy logic principles. The Sugeno fuzzy model-based Adaptive Neuro-fuzzy system had been used to classify seven distinct movements in a longer test duration lasting for about three hours, achieving an average accuracy of 86% based on TD features [13]. In 2011, a study on real-time intelligent pattern recognition algorithms for surface EMG signals provided 97% average accuracy on the discrimination of six classes of hand movements using the ANFIS approach based on TFD features [94].

The SVM is a kernel-based approach and has become an increasingly popular tool for machine learning tasks involving classification and regression. SVM which is a promising data classification technique proposed by Vapnik [96] is generated from the training process using the training data. Later on, classification is implemented based on the trained model. The main problems encountered in setting up the SVM model are how to decide on the kernel function and its parameter values. In aiming for multiple users to perform multiple motions, the bilinear model is proposed by composing two linear factors that are user dependent and motion dependent to classify five hand gestures using SVM [34]. This method resulted in 73% accuracy, meanwhile, 96.75% [22] were gained by hybridizing the particle swarm optimization (PSO) and SVM in detecting neuromuscular disorders. These findings reported that the kernel parameter setting of SVMs in EMG signal classification based on TFD features (DWT) affects classification performance.

### 4.5. EMG Evaluation Metrics

In this section, an approach to determine the classification accuracy of proposed systems by previous researchers will be identified. Different approaches have been used to evaluate the systems. Moreover, the purpose of each study and feature extracted will also be explained.

In order to develop a system that characterizes certain movements of the human arm, Balbinot and Favieiro [13] extracted the TD features (RMS) of surface EMG signals recorded in a long duration at approximately three hours. The pattern of arm movements were classified using ANFIS. As a result, their system obtained an average accuracy of 86% based on the desired output, animations of the virtual model which appear on the LCD screen with real output, while the hand movements of the subject were compared.

After this study, Phinyomark et al. [27] compared the stability of a single feature and multiple feature sets within 21 days by observing the behavior of fifty TD and FD features to classify ten upper limb motions. Based on training and testing the EMG data, multiple features gained higher classification accuracy at 98.87% compared to the single feature using LDA as a classifier. All experiments used the EMG trials (i = 2–121) as the testing set, whereas the training set trials are different in each experiment: (1) training on the initial data (only the first day); (2) training on the most recent data (five recent preceding trials); and (3) all preceding data.

Similarly, motion recognition of one subject is assigned as a testing data and the remaining data are assigned as training data to construct the bilinear model (TD features) in designing an EMG interface that can be used by multiple users to perform multiple motions [34]. In their study, the average classification accuracy were defined as $E=N_{correct}/N$, where $N_{correct}$ is the number of correctly classified samples and N is the size of the test samples which is 11. As their method resulted in 73% accuracy, it can be conclude that it was difficult to perform multiple motions based on multichannel of EMG signals.

Using the same theory of accuracy, Ahmad considered the accuracy in percentage as shown in Equation (11) [35]. The FL system had successfully classified the stages of contraction (e.g., start, middle and end) on wrist muscles with 97% accuracy.

(10) $$Accuracy=\frac{\mathrm{number}\;\mathrm{of}\;\mathrm{correct}\;\mathrm{classification}}{\mathrm{number}\;\mathrm{of}\;\mathrm{total}\;\mathrm{classifications}}\times 100$$

In an assessment of muscle fatigue, tracking of muscle activity in isotonic and isometric contractions was investigated by Rogers et al. [23]. However, they considered isotonic contractions at instances when the angle during elbow flexion and extension is between 50 and 130. This technique is inappropriate as isotonic contractions occur when the length of muscle changes as mentioned in Section 2. Instead of extracting the features of EMG signals in isotonic conditions, Subasi [22] diagnosed neuromuscular disorders such as normal, neurogenic or myopathic using hybridized PSO-SVM classification based on TFD features in isometric conditions. In their work, the number of true positives (TP), false positives (FP), true negatives (TN), and false negatives (FN) are used in evaluating the performance of a classifier. Different terms are used in different domains. The sensitivity and specificity refers to the proportion of people with disease who has a positive test result to the proportion of people without disease who has a negative test result, which is 1-FP respectively. The sensitivity and specififcity are defined as belows:

(11) $$Sensitivity=\frac{TP}{TP+FN}\times 100$$

(12) $$Specificity=\frac{TN}{TN+FP}\times 100$$

To calculate the accuracy, an overall measure are implemented, which is:

(13) $$Accuracy=\frac{Sensitivity+Specificity}{2}\times 100$$

Even though the study obtained 96.75% accuracy, as noted by Li et al. [54] the power spectral analysis can serve as a useful tool to detect neural and muscular changes. The neural change in normal gaits during isotonic conditions were explored on by Ogawa et al. [97] to enhance passive locomotor-like movements when accompanied by arm swing movements. Despite that, Ahsan et al. [47] and Ibrahimy et al. [98] considered the number of input features, hidden neurons, learning algorithms, correlation between network outputs and targets, and mean square error to analyze the ANN performances of different architectures of neural network as shown in Table 6. Ahsan et al. and Ibrahimy et al. discovered that features extracted using TD features achieved 89.2% and 88.4% of accuracy, respectively.

A number of authors have considered training and validation of data to determine the effectiveness of their system [29,44,94]. Tsai et al. divided the recognition experiments into training and validation phases to compare the differences of upper limb motion patterns during dynamic and isometric muscle contractions with four conditions: (1) training and validation phase data containing EMG signals with dynamic contractions, denoted as “D-D”; (2) training and validation phase data containing EMG signals with isometric contractions, denoted as “I-I”; (3) training data of dynamic contractions, and validation data of isometric contractions, denoted as “D-I”; and (4) training data of isometric contractions, and validation data of dynamic contractions, denoted as “I-D” [29]. The results of their experiment presented STFT-ranking feature exceeds 90% of recognition accuracy during the training and validation phases of the same type muscle contractions.

The work done by Englehart et al. divided EMG data into a training set (100 patterns), a test set (150 patterns), and a validation set (150 patterns) to compare the performance of TD and TFD features. The validation set provides an estimate of the classification performance of the test set [44]. Meanwhile, Khezri and Jahed [94] considered 100 signals for each class to extract the feature set from four different subjects and divided these signals into two groups, namely 50 signals as the training set and 50 signals for the test set. For the training set, arbitrary outputs were chosen for each hand motion. They were able to determine the correctness of the system by recognizing each hand motion after training it using the three pattern-recognition systems and obtaining the estimated outputs for each class. Overall, all studies discussed in this section are summarized in Table 7.

## 5. EMG Dataset

### 5.1. Placement of Electrodes

This section will briefly explain the suitable muscle that has been considered by previous researchers. For upper limb movement, most studies conducted have characterized hand and arm movements based on EMG signals recorded on the biceps brachii muscle during isometric contractions [65,76,99]. Later, biceps brachii and triceps brachii muscles were selected in the analysis of motion pattern EMG signals in isotonic and isomteric contractions [29]. Balbinot and Favieiro had interpreted EMG signals recorded from an 8-channel located on the biceps, palamaris longus, flexor carpi ulnaris, flexor carpi radialis, pronator teres, extensor digitorum, brachio radialis and extensor carpi ulnaris muscles to characterize arm movements [13]. Besides that, Matsubara and Morimoto extracted information on EMG signals from the flexor carpi ulnaris, extensor carpi radialis, flexor digitorum profundus and extensor digitorum muscles in their study [34]. It is similar with the work done by Ahmad and Chappell [12] who detected the stages of contraction of wrist muscles.

There are relatively few historical studies in the area of analysis of EMG signals on lower limbs. Instead of classifying motion patterns of EMG signals, lower limb applications are more related in clinical practice and EMG signals were collected during isotonic contractions. Walking pattern has become the main concern in a majority of studies in lower limb applications. To illustrate walking patterns, the tibialis anterior (TA), gastrocnemius lateralis, gastrocnemius medialis, soleus and flexor hallucis longus muscles were selected [100]. To discriminate walking speeds, a study conducted in [101,102,103] and Hussain et al. [104] extracted EMG signals from the rectus femoris (RF) muscle. Meanwhile, Ogawa et al. detected normal gaits by placing electrodes on TA, medial head of the gastrocnemius, RF, biceps femoris, anterior head of deltoid (aDEL), and the posterior head of deltoid (pDEL) muscles [97].

### 5.2. Muscle Conditions

Several recent studies have investigated motion patterns of EMG signals in isotonic and isometric conditions. Generally, raw data of EMG signals in isometric contractions as reported by Daud et al. [7] within hand-lifting of three different loads: (a) 1 kg; (b) 3 kg; (c) 5 kg. In 2011, Lorrain et al. [26] recorded EMG signals of 9 hand motions in 10 s duration with 3 s resting periods between consecutive contractions of 8 subjects. In each contraction, the subject was instructed to start from the rest position and reach the target position in 3 s, then maintain the target position for 4 s, and return to the rest position in 3 s. They segment of static portion (4 s in the middle), and two segments of dynamic (anisotonic and anisometric, representing the two main dynamic situations in real movements) portion (3 s at each end) in each contraction.

In 2013, a study conducted by Phinyomark et al. instructed subjects to perform eleven upper limb motions including extension (m1), flexion (m2), ulnar deviation (m3), radial deviation (m4), pronation (m5), supination (m6), open (m7), close (m8), key grip (m9), pincer grip (m10), and extract index finger (m11) for five seconds [27]. After this study, a comparison of upper limb motion pattern of EMG signals in both conditions was revealed by Tsai et al. [29]. Based on the experimental results, the same type of muscle contraction is preferable to be used in training and validation phases in determining the motion pattern recognition performance.

## 6. Discussion

This study described the importance of pattern recognition methods of EMG signals, which is very important in many applications, such as rehabilitation devices, prostheses, orthoses and detection for neuromuscular disorders. As EMG signals contain undesired signal sources such as ECG artifacts, filtering techniques is suggested to eliminate all the noises. However, this process may reduce the noises but the quality of EMG signals is not guaranteed. For real-time control of a robotic arm or leg, the classification of EMG signals is an important issue. Therefore, researchers tend to focus on processing methods of EMG signals in order to obtain a more accurate, simple and reliable system in detecting the motion patterns. It is noted that a large number of EMG channels will increase the number of control commands of the classifier, thus effecting the computational time. For feature extraction, TD and TFD features are widely used in the literature compared to FD features as performance using FD features is not promising, especially in isometric conditions. In the case of upper limb motions, the performance of TFD features using ANFIS and SVM classifications are better than TD features based on the classification accuracy. Nonetheless, higher accuracy was gained using TD features with LDA as a classifier than TFD features in order to characterize hand movements. LDA methods are recommended when a huge number of features are used as input to the classifier. Even when the results are obtained with different types of muscle contractions, it is preferred by previous researchers to analyse EMG signals in same muscle contractions.

On the other hand, the new approach, PDF is recommended as a feature to be extracted in motion pattern recognition. The main advantage of this approach is its mathematical modelling in understanding the intrinsic biological phenomenon of EMG signals, improving the algorithm for onset muscle activation and is a useful tool to indicate muscular changes. Based on reviews, the potential of PDF in describing EMG signals for isometric contractions are for a Gaussian distribution at higher level contractions and a Laplace distribution at low level contractions. Meanwhile, there is little attention in the case of lower limb motion as the analysis of leg movement is more related to clinical practice. Walking patterns have become the interest of researchers in most studies, such as walking patterns at different speed of subjects with and without neuromuscular diseases such as cerebral palsy. To increase classification accuracy, a combination of processing methods and pattern recognition techniques along with similar muscle contractions is strongly suggested. This combination method may be helpful to increase classification accuracy without having to use too many muscle positions.

## 7. Future Trends

Other important findings that should be researched upon in the future to improve the development of control system performance are as recommended below:

- Most studies do not critically highlight the pre-processing stage. To remove the artifacts in EMG signals, the cutoff frequency should be in the range between 20 Hz to 500 Hz and a Butterworth filter commonly applied. Meanwhile, the segmentation techniques used should depend upon its applications. However, the comparison performance between adjacent and overlapped windowing technique has not yet been identified.
- Feature extraction is the most difficult part in motion pattern recognition. In the literature, they compared the performance of TD, FD and TFD features of EMG signals during isometric contractions. The features of muscle contractions under isotonic has yet to be explored.
- Sample data of EMG signals has always been fitted with PDF under physical situations which correspond to non-fatiguing conditions, also called as isometric contractions. However, the PDF of isotonic contractions for EMG signals has never been investigated.
- To achieve better classification accuracy, significant features extracted is the main contribution. A classifier can be chosen depending on the number of features. Different classifiers result in different percentage error.
- Pattern recognition of EMG signals for upper limbs has been widely investigated compared to lower limbs especially in isometric contraction. The proper isotonic contractions behind the generation of the EMG signals is still unknown.

## 8. Conclusions

This paper provides an overview of how EMG signal systems are designed, in particular on controlling the HMI for improving the quality of life for individuals. Several challenge issues referring to an analysis of EMG signals are such as the signals are non-deterministic. Recent advances in the analysis of EMG signal studies that are gathered and reviewed include areas of pre-processing, feature extraction, PDF and performance of features to classify motion pattern recognition with different conditions of muscles. From the reviews that were made, a conclusion can be drawn that continued efforts are needed to make EMG signals more accessible in medical and physiological applications, especially during isotonic and isometrics conditions. Moreover, interpretation regarding both contractions becomes much easier for the development of assistive devices.

## Figures and Tables

**Table 1:** Time domain features.

Features	Abbreviation	References
Integrated EMG	IEMG	[28]
Mean Absolute Value	MAV	[7,28,42,47,50]
Modified mean absolute value 1	MAV1	[28,51]
Modified mean absolute value 2	MAV2	[28,51]
Root Mean Square	RMS	[7,13,28,47]
Variance	VAR	[28,47]
Waveform length	WL	[28,42,50]
Zero crossing	ZC	[28,42,50]
Slope sign change	SSC	[28,42,47]
Willison amplitude or Wilson amplitude	WAMP	[28,47]
Kurtosis	KURT	[31]
Skewness	SKEW	[52]
Moving Approximate Entropy	moving ApEn	[35]
Fuzzy approximate entropy	fApEn	[48]
Simple square integral	SSI	[28]
v-Order	V	[28,50]
Log detector	LOG	[28]
Average amplitude change	AAC	[28]
Difference absolute standard deviation value	DASDV	[28]
Mean absolute value slope	MAVSLP	[28]
Multiple hamming windows	MHW	[28]
Multiple trapezoidal windows	MTW	[28]
Histogram of EMG	HIST	[50]
Auto-regressive coefficients	AR	[50]
Cepstral coefficients		[28]
Standard deviation	SD	[7,42,47]
Cepstral coefficients	CC	[28]
Sample entropy	SampEn	[53]
Integral absolute value	IAV	[50]
Variance	VAR	[50]
Maximum amplitude	MAX	[7]

**Table 2:** Frequency domain features.

Features	Abbreviation	References
Mean frequency	MNF	[28,41]
Median frequency	MDF	[28,41]
Mean power frequency	MNP	[51]
Peak frequency	PKF	[28]
Total power	TTP	[28]
Frequency ratio	FR	[28]
Power spectrum ratio	PSR	[28]
The power spectrum deformation	Ω	[41]
Variance of central frequency	VCF	[28]
Signal-to-motion artifact ratio	SMR	[41]
Signal-to-noise ratio	SNR	[41]
Spectral moment	SM	[28]
Energy	EN	[42]
Wavelet decomposition	WDC	[42]
Wavelet decomposition difference	WDCDIF	[42]
Modified mean frequency	MMNF	[56]
Modified median frequencies	MMDF	[56]
Short Time Fourier transform	STFT	[57]

**Table 3:** Time Frequency domain features.

Features	Abbreviation	References
Discrete Wavelet Transform	DWT	[44]
Continous Wavelet Transform	CWT	[9]
Empirical Mode Decomposition	EMD	[9]
Wavelet Packet Transform	WPT	[44]

**Table 4:** The mean of the MAE features of each theoretical PDF for each percentile load level condition.

PDF	Maximum Voluntary Contraction				
	20%	40%	60%	80%	100%
Normal	0.0036	0.0025	0.0024	0.0022	0.0028
Laplace	0.0081	0.0075	0.0076	0.0077	0.0071
Cauchy	0.0129	0.0123	0.0123	0.0124	0.0122
Logistic	0.0027	0.0012	0.0009	0.0011	0.0015

**Table 5:** Comparison PDF of EMG signals.

PDF	Authors
Gaussian	[43,76,77,78,85]
Cauchy	[65]
Laplace	[76,77]
Logistic	[65]
GEV	[81]

**Table 6:** The classification accuracy based on training functions [98].

Training	Stop	Regression	Time	Classification Rate				Hidden
Function	Epochs		Elapsed	Training	Validation	Test	Overall	Neurons
Levenberg marquardt	15	0.8597	1.047	88.6	83.3	90	88	10
	18	0.87251	0.921	94.3	66.7	80	88	
	16	0.87401	0.8721	88.7	90.3	90.3	89.2	
	Average	0.86874	0.947	90.533	80.1	86.767	88.4	
	33	0.85706	2.797	91.4	70	83.3	87	20
	14	0.85508	1.218	90	80	86.7	88	
	12	0.84772	1.094	92.9	76.7	83.3	89	
	Average	0.853287	1.703	91.433	75.567	84.433	88	
	16	0.86112	2.36	92.1	80	76.7	88	30
	11	0.85018	1.703	91.4	90	73.3	88.5	
	14	0.85192	2.125	89.3	76.7	83.3	86.5	
	Average	0.854107	2.0627	90.933	82.233	77.767	87.667	
Scaled Conjugate Gradient	37	0.7819	0.703	80.7	83.3	83.3	82.43	10
	27	0.7632	0.685	78.2	86	74.5	79.57	
	32	0.7904	0.823	82.4	71.9	79.4	77.9	
	Average	0.77917	0.737	80.433	80.4	79.067	79.9	
	31	0.802	0.797	78.6	90	82.7	83.77	20
	35	0.8153	1.252	79	87.3	78.1	81.47	
	34	0.79842	1.063	84.3	76.7	80	80.33	
	Average	0.80524	1.037	80.633	84.667	80.267	81.86	
	34	0.80767	2.457	83.6	83.3	86.7	84.53	30
	28	0.79215	1.073	81.2	72.1	69.5	74.27	
	31	0.82531	1.352	86.6	76.5	78.8	80.63	
	Average	0.80837	1.627	80.433	80.4	79.067	79.9	

**Table 7:** Comparison of classification accuracy.

Authors	Feature Extraction	Classifier	Accuracy
[13]	TD	ANFIS	86%
[27]	TD	LDA	98.87%
[34]	TD	SVM	73%
[28]	TD	LDA	91.64 %
[35]	TD	FL	97%
[47]	TD	ANN	89.2%
[29]	TFD	SVM	90%
[44]	TFD	LDA	93.75%
[94]	TFD	ANFIS	92%
[98]	TD	ANN	88.4%
[22]	TFD	PSO-SVM	96.75%
